# Newborn Weight Loss as a Predictor of Persistence of Exclusive Breastfeeding up to 6 Months

**DOI:** 10.3389/fped.2022.871595

**Published:** 2022-04-07

**Authors:** Enrica Delfino, Luca Peano, Roberto Giorgio Wetzl, Maria Lorella Giannì, Roberta Netto, Alessandra Consales, Maria Enrica Bettinelli, Daniela Morniroli, Francesca Vielmi, Fabio Mosca, Luca Montagnani

**Affiliations:** ^1^Department of Anesthesia, Intensive Care, and Out-Hospital Emergency, Ospedale Regionale della Valle d'Aosta, Aosta, Italy; ^2^Department of Pediatrics, Ospedale Regionale della Valle d'Aosta, Aosta, Italy; ^3^Department of Clinical Sciences and Community Health, University of Milan, Milan, Italy; ^4^Fondazione IRCCS Ca' Granda Ospedale Maggiore Policlinico, Neonatal Intensive Care Unit, Milan, Italy

**Keywords:** newborn weight loss, weight loss up to 7%, weight loss higher than 7%, exclusive breastfeeding up to 6 months, integration with formula, complementary feeding, exclusive formula feeding, weaning

## Abstract

**Objectives:**

To investigate the association between neonatal weight loss and persistence of exclusive breastfeeding up to 6 months.

**Study Design:**

An observational cohort study in the setting of a Baby Friendly Hospital, enrolling 1,260 healthy term dyads. Neonatal percentage of weight loss was collected between 48 and 72 h from birth. Using a questionnaire, all mothers were asked on the phone what the infant's mode of feeding at 10 days, 42 days and 6 months (≥183 days) from birth were. The persistence of exclusive breastfeeding up to 6 months and the occurrence of each event that led to the interruption of exclusive breastfeeding were verified through a logistic analysis that included 40 confounders.

**Results:**

Infants with a weight loss ≥7% were exclusively breastfed at 6 months in a significantly lower percentage of cases than infants with a weight loss <7% (95% CI 0.563 to 0.734, *p* < 0.001). Weight loss ≥7% significantly increases the occurrence of either sporadic integration with formula milk (95% CI 0.589 to 0.836, *p* < 0.001), complementary feeding (95% CI 0.460 to 0.713, *p* < 0.001), exclusive formula feeding (95% CI 0.587 to 0.967, *p* < 0.001) or weaning (95% CI 0.692 to 0.912, *p* = 0.02) through the first 6 months of life.

**Conclusions:**

With the limitations of a single-center study, a weight loss ≥7% in the first 72 h after birth appears to be a predictor of an early interruption of exclusive breastfeeding before the recommended 6 months in healthy term exclusively breastfed newborns.

## Introduction

An ever-growing body of evidence indicates that exclusive breastfeeding for the first 6 months must be considered a public health priority, since it promotes maternal-infant well-being and reduces healthcare costs. The World Health Organization (WHO) and the United Nations Children's Fund (UNICEF) strongly recommend exclusive breastfeeding for 6 months after delivery, followed by continued breastfeeding during weaning. However, breastfeeding rates remain low, settling in Europe between 13 and 39% (1).

Breastfeeding is a complex process, whose achievement implies the successful interaction between two individuals. It requires that both the mother and the newborn have an intact neurological status, a good state of health, and the ability to interact with each other. None of the methods currently used to assess the well-being of the newborn (e.g., Apgar score or neurobehavioral tests) is predictive of the success of this interaction, and many maternal variables, including age, parity, mode of delivery, infusion of intravenous fluids during delivery, attachment style (2–4), may interfere with it.

The currently available literature suggests that the second and third days following birth (48–72 h post-partum) appear to be the days of maximum weight loss (5). Postnatal weight loss up to 7% at nadir can be considered physiological (6, 7). It must be taken into consideration that the extent of postnatal weight loss is affected by many variables. On the basis of the available evidence, clinical guidelines caution that a postnatal weight loss greater than 7% indicates the need for careful assessment of the neonate and could indicate ineffective breastfeeding (6). However, to the best of our knowledge, to date no data are available on the predictive value of the magnitude of postnatal weight loss on exclusive breastfeeding continuation up to 6 months. The aim of this study was to investigate differences between full-term newborn infants showing a Weight Loss (WL) <7 or WL ≥7% at nadir both in terms of persistence of exclusive breastfeeding at 6 months and occurrence of events leading to its interruption (any integration, complementary feeding, shift to exclusive formula feeding, weaning).

## Materials and Methods

This manuscript adheres to the Strengthening the Reporting of Observational Studies in Epidemiology guidelines. This study project was reviewed and approved by the Institutional Ethics Committee (register 166798) of the Parini Regional Hospital, which is the only facility in Aosta Valley and, since 2010, has been repeatedly designated by UNICEF as a Baby-Friendly Hospital.

At our hospital, every mother receives standardized breastfeeding support, regardless of type of delivery (vaginal delivery or cesarean delivery) or labor analgesia. The decision to administer formula milk or glucose to newborns follows extremely strict criteria, in line with the recommendations of the WHO/UNICEF Ten Steps to Successful Breastfeeding. Infants are weighed between 2 and 6 h from birth and between 48 and 72 h from birth (at discharge) and WL is expressed as a percentage of birthweight, calculated on the lowest weight reached.

We conducted an observational cohort study, from January 1st, 2014 to September 22nd, 2015.

For the purposes of the present study, we considered eligible mothers of healthy newborns (to avoid bias related to underlying diseases), born at term (i.e., from 37 to 41 + 6 weeks gestational age) and with a birthweight between 2,500 and 4,000 g. We decided to exclude preterm and small for gestational age newborns, since they have been reported to be at increased risk for greater postnatal weight loss than full-term and appropriate for gestational age ones (8–10). Other infants' health conditions arisen after birth did not exclude newborns from eligibility but were considered as confounders. Newborns from single mothers were excluded because fathers' support can influence mothers' breastfeeding decisions and behavior (11, 12). Furthermore, several confounders considered in the analyses were relevant to both parents as a couple.

Lastly, we excluded women who asked for epidural analgesia for pain relief during labor and did not receive it due to technical problems. Indeed, it is not clear whether labor analgesia's repercussions on breastfeeding are related to the technique itself or to the conditions that lead to require it: the choice itself to request labor analgesia could be linked to difficulties in breastfeeding (13–16).

Maternal health conditions were not included in the exclusion or inclusion criteria but were considered as confounders.

Among those considered eligible at birth (*n* = 1834), at the time of discharge we enrolled 1,260 consecutive dyads: we recruited only those who were exclusively breastfeeding at 72 h from birth (in the case of twins, we considered the first born), and, according to the WHO definition of exclusive breastfeeding (17), did not receive formula milk or any other fluid during hospital stay, except for medicines, vitamins or minerals. Birth weight was collected between 2 and 6 h from birth, and at the nadir of weight loss, that is, between 48 and 72 h from birth (at discharge). On the basis of the calculated percentage of weight loss (WL) at nadir, newborns were subsequently divided into two groups: WL <7 and WL ≥7%.

The confounders that, according to the current literature, could be associated with early interruption of exclusive breastfeeding (18, 19) were entered into the analysis. Altogether, we identified 40 confounders, grouped as 23 antelabor and 17 peripartum confounders (Table 1), where peripartum confounders included intrapartum and postpartum ones. To be considered antelabor, a confounder had to: be present before labor initiation, have an alleged association with the endpoint (persistence of breastfeeding up to 6 months), and belong to a temporal sequence impossible to reverse.

**Table 1:** Antelabor and peripartum confounders analyzed, and number of missing values.

	**Total population (** ***n*** **)**			**Weight loss** ** <7% (*****n*****)**			**Weight loss** **≥7% (*****n*****)**		
	**Yes**	**No**	**Missing**	**Yes**	**No**	**Missing**	**Yes**	**No**	**Missing**
**Antelabor confounders**									
Maternal age <10th centile	80	1,191		59	814		21	377	
Maternal age >90th centile	102	1,169		60	813		42	356	
Paternal age <10th centile	114	1,146	11	83	784	6	31	362	5
Paternal age >90th centile	98	1,162	11	65	802	6	33	360	5
Mixed couple	120	1,151		80	793		40	358	
Foreign couple	171	1,100		127	746		44	354	
Lower maternal education	280	991		199	674		81	317	
Lower paternal education	441	830		306	567		135	263	
In-country couple residence	1,146	125		792	81		354	44	
Independent mother's income	966	305		646	227		320	78	
Mother herself breastfed	927	342	2	641	230	2	286	112	
Breast problems	15	1,256		9	864		6	392	
Maternal BMI <10th centile	125	1,146		85	788		40	358	
Maternal BMI >90th centile	112	1,159		69	804		43	355	
Smoking during pregnancy	116	1,155		87	786		29	369	
Pregestational dysthyroidism	97	1,174		61	812		36	362	
Severe pre-eclampsia	21	1,250		15	858		6	392	
Maternal diabetes	107	1,164		69	804		38	360	
Maternal arterial hypertension	46	1,225		29	844		17	381	
Other maternal diseases	68	1,203		40	833		28	370	
Assisted reproductive procedures	27	1,244		12	861		15	383	
Nulliparity/primiparity	625	646		389	484		236	162	
Gestational hypothyroidism	42	1,229		27	846		15	383	
**Peripartum Confounders**									
Labor analgesia	223	1,048		155	718		68	330	
Cesarean delivery	333	938		181	692		152	246	
Weight gain during pregnancy <10th centile	72	1,199		55	818		17	381	
Weight gain during pregnancy >90th centile	131	1,140		81	792		50	348	
Male newborn sex	671	600		462	411		209	189	
Post partum hemorrhage >1,000 ml	52	1,219		31	842		21	377	
Peripartum interventions	110	1,161		69	804		41	357	
Meconium-stained amniotic fluid	396	875		287	586		109	289	
APGAR score at 5' <7	6	1,265		2	871		4	394	
Neonatal septic risk	102	1,169		72	801		30	368	
Neonatal hypoglycaemia	43	1,228		25	848		18	380	
Neonatal phototherapy	26	1,245		16	857		10	388	
NICU admission	27	1,244		19	854		8	390	
Skin-to-skin contact	987	284		696	177		291	107	
Rooming-in	1,076	195		749	124		327	71	

*Maternal age: 10th centile = 24 years, 90th centile = 39 years; paternal age: 10th centile = 28 years, 90th centile = 44 years; maternal BMI: 10th centile = 18.5, 90th centile = 28.4; lower maternal education = lower than 13 years of school education; lower paternal education=lower than 13 years of school education; neonatal septic risk: determined by the coexistence of the following risk factors: newborn with early-onset Group B Streptococcal sepsis in a previous pregnancy, maternal antepartum temperature ≥38°C, maternal chorioamnionitis, prolonged rupture of membranes >18 h, maternal Group B Streptococcal positive status, broad spectrum antibiotics administered >4h prior to birth; neonatal hypoglycemia: plasma glucose level <30 mg/dl in the first 24 h of life; rooming-in: the baby remains with the mother 24 h a day for the entire duration of hospital stay*.

Data were obtained from the database filled by the hospital's midwives assisting childbirth.

In most cases, previously published and validated cut-offs were used. If the percentile cut-off was used, the 10th and 90th percentiles were calculated for the entire population of women who delivered at our facility.

Our main endpoint was the duration of exclusive breastfeeding defined according to the WHO definition. The secondary endpoint was the occurrence of each event that led to the interruption of exclusive breastfeeding, according to the WHO definition: sporadic integration with formula milk (day on which an integration, even single, with formula milk occurs for the first time), complementary feeding (day on which breast milk begins to be supplemented daily with formula milk), shift to exclusive formula feeding (day on which exclusive formula feeding is started), and weaning (day on which any food other than breast milk or formula milk is introduced, including fruit).

After discharge, all mothers were reached on the phone by an anesthesiologist skilled in obstetric anesthesia and an International Board Certified Lactation Consultant (IBCLC) himself, at 10 days, 42 days and 6 months (≥183 days) from birth. Mothers were asked about what the infant's mode of feeding during the previous week had been, and time of introduction of liquids other than breast milk, or weaning. Mothers were asked the exact date on which the events under examination occurred, and these dates were reported in the database as follows: time of interruption of exclusive breastfeeding (identified as the time of occurrence of sporadic integration), start of complementary feeding, start of exclusive formula feeding, weaning.

Newborns were considered lost to follow-up if mothers could not be reached on the phone at any time point.

### Statistical Analysis

Data was collected and tabulated using the File-Maker 11 Pro Relational Database (FileMaker, FileMaker International, Santa Clara, California, USA). Statistical analyses were performed using SPSS V.22 (IBM SPSS Statistics).

The analysis was performed with the absolute frequencies for qualitative variables, and with the median and the interquartile range for quantitative variables. If a categorical variable had three or more possible values, dummy variables were created.

Quantitative variables, i.e., age, Body Mass Index (BMI), weight loss, were handled according to a percentile distribution.

To verify whether there was a statistically significant difference in the primary endpoint (i.e., exclusive breastfeeding in the first 6 months of the infant's life) between the groups WL <7 and WL ≥7%, we examined a 2x2 contingency table. The result obtained was verified through a logistic analysis that included all the available antelabor and peripartum confounders. We performed the same statistical analysis for the occurrence of integration, complementary feeding, shift to formula feeding, and weaning.

Finally, with a Cox regression, we verified the temporal trend of both exclusive breastfeeding and the four events that determined its interruption in the two groups, considering all confounders.

## Results

**Figure 1:**
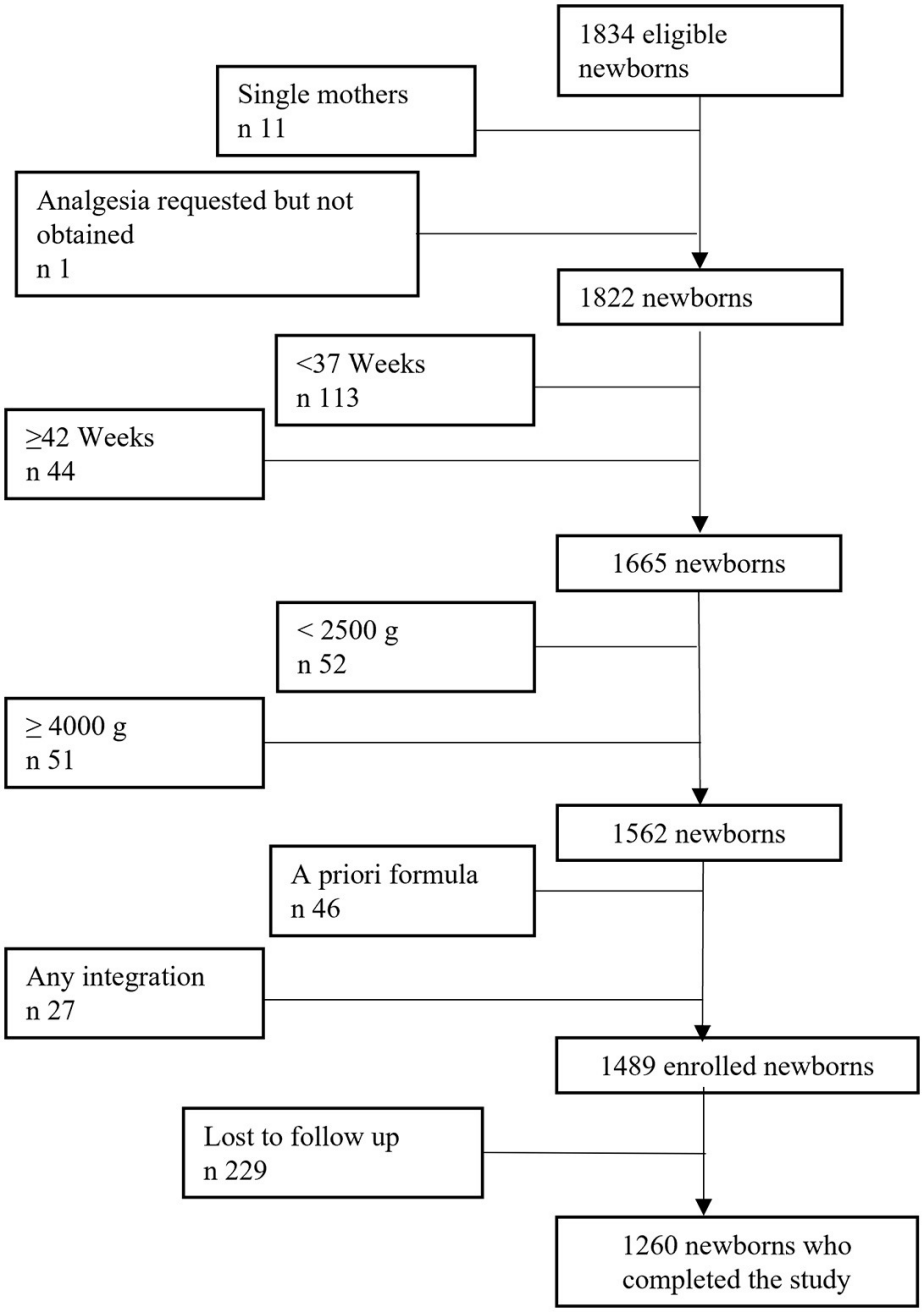
Dyads' flow through the study.

Figure 1 shows dyads' flow through the study. No differences in basal characteristics were found between mothers who were excluded from the study and those who were included (data not shown).

In our study population 68.7% of newborns lost <7% of birth weight at 72 h and mean WL at 72 h was 5.8% (+/−2.29). The WL <7% group lost on average 4.6% (+/−1.62) and the WL ≥7% group lost on average 8.4% (+/−1.07) of birth weight. As for mode of delivery, newborns born through cesarean delivery lost significantly more weight than newborns born through vaginal delivery (6.18 vs. 5.39%, respectively, *p* <0.001).

Table 1 lists antelabor and peripartum confounders. Missing data are declared variable by variable.

As demonstrated by Pearson chi-square test (Table 2), infants with WL ≥7% were exclusively breastfed at 6 months (day 183) from birth in a significantly lower percentage of cases than infants with WL <7% (*p* ≤ 0.001) whereas they received either sporadic integration with formula milk, or complementary feeding, or exclusive formula feeding, or were weaned through the first 6 months of life in a significantly higher percentage of cases.

**Table 2:** Pearson chi-square test.

			**Exclusive breastfeeding**		
			YES	NO	Total
Weight Loss ≥7%	NO	Number	131	736	867
		%	15.1%	84.9%	100.0%
	YES	Number	28	365	393
		%	7.1%	92.2%	100.0%
Total population		Number	159	1,101	1,260
		%	12.6%	87.4%	100.0%
	**Value**	**Dof**	**Significance**		
Pearson χ^2^	15.636	1	0.000		
	***Integration***				
			*NO*	*YES*	*Total*
Weight Loss ≥7%	*NO*	*Number*	*494*	*373*	*867*
		*%*	*57.0%*	*43.0%*	*100.0%*
	*YES*	*Number*	*137*	*256*	*393*
		*%*	*34.9%*	*65·1%*	*100.0%*
Total population		*Number*	*631*	*629*	*1,260*
		*%*	*50.1%*	*49.9%*	*100.0%*
	***Value***	**Dof**	***Significance***		
Pearson χ2	*52.917*	*1*	*0.000*
	**Complementary feeding**				
			NO	YES	Total
Weight Loss ≥7%	NO	Number	626	241	867
		%	72.2%	27.8%	100.0%
	YES	Number	216	177	393
		%	55.0%	45·0%	100.0%
Total population		Number	842	418	1,260
		%	66.8%	33.2%	100.0%
	**Value**	**Dof**	**Significance**
Pearson χ2	36.260	1	0.000
	**Exclusive formula feeding**				
			NO	YES	Total
Weight Loss ≥7%	NO	Number	683	184	867
		%	78.8%	21.2%	100.0%
	YES	Number	259	134	393
		%	65.9%	34·1%	100.0%
Total population		Number	942	318	1260
		%	74.8%	25.2%	100.0%
	**Value**	**Dof**	**Significance**		
Pearson χ2	23.754	1	0.000
	**Weaning**				
			NO	YES	Total
Weight Loss ≥7%	NO	Number	180	687	867
		%	20.8%	79.2%	100.%
	YES	Number	60	333	393
		%	715.3%	84.7%	100.0%
Total population		Number	240	1,020	1,260
		%	19.0%	81.0%	100.0%
	**Value**	**Dof**	**Significance**		
Pearson χ2	5.294	1	0.021		

*Persistence of exclusive breastfeeding and occurrence of integration, complementary feeding, exclusive formula feeding and weaning at 6 months of the infant's life. The number of infants still breastfed at 6 months and the number of infants in whom each event occurred within the first 6 months is indicated for each of the two groups under examination. Dof, degrees of freedom*.

Both the interruption of exclusive breastfeeding and each of the four events that determined it, occurred at a significantly earlier age in the WL ≥7% group than in the WL <7% group (Table 3).

**Table 3:** Test of the median with independent samples median test.

		**Exclusive breastfeeding at 6 months**	**Sporadic integration**	**Complementary feeding**	**Exclusive formula feeding**	**Weaning**
Total population	n	1,101	629	418	318	1,260
	Median	124	23	30	92	167
	25th centile	14	3	9	43	152
	75th centile	162	117	122	129	177
Weight Loss <7%	n	736	373	241	184	867
	Median	145	36	40	93	168
	25th centile	35	10	14	61	153
	75th centile	167	122	122	137	179
Weight Loss ≥7%	n	365	256	177	134	393
	Median	34	9	3	77	163
	25th centile	3	3	12	32	146
	75th centile	152	43	53	122	176
Statistical significance (*p*)		**0.000**	**0.000**	**0.000**	**0.050**	**0.000**

*For each event, and for each group under consideration, the median of the day after birth in which it occurred (and the day at 25th and at 75th percentile) is indicated*.

*Values in bold are p-values: infants with weight loss > 7% receive supplementation, complementary feeding, exclusive formula and weaning significantly earlier*.

The logistic regression confirmed the association between WL ≥7% and the interruption of exclusive breastfeeding before 6 months, reaching statistical significance on all considered outcomes [exclusive breastfeeding at day 183 from birth (95% CI 0.563 to 0.734, *p* < 0.001), sporadic integration with formula milk (95% CI 0.589 to 0.836, *p* < 0.001), start of complementary feeding (95% CI 0.460 to 0.713, *p* < 0.001), start of exclusive formula feeding (95% CI 0.587 to 0.967, *p* = 0.02), start of weaning (95% CI 0.692 to 0.912, *p* = 0.001)]. Among the confounders, no one was associated at the same time with all four outcomes (Table 4; relative risk, confidence interval and statistical significance are shown in Tables 5, 6).

**Table 4:** Summary table of statistical significance.

	**Exclusive breastfeeding at 6 months**	**Integration with formula**	**Introduction of formula**	**Exclusive formula feeding**	**Weaning**
	**Effect**	**Effect**	**Effect**	**Effect**	**Effect**
**Antelabor confounders**					
Maternal age <10th centile	**↑**	**↑**	**↑**	**↑**	**↑**
Maternal age >90th centile	**↑**	**↑**	**↑**	**↓**	**↓**
Paternal age <10th centile	**↑**	**↑**	**↑**	**↑**	**↑**
Paternal age >90th centile	**↑**	**↑**	**↑**	**↑**	**↓**
Mixed couple	**↓**	**↑**	**↑**	**↓**	**↑**
Foreign couple	**↑**	↓^*^	**↓**	**↓**	*↓
Lower maternal education	**↓**	**↑**	**↑**	**↑**	**↓**
Lower paternal education	**↓**	**↑**	**↑**	**↑**	**↑**
In-country couple residence	**↑**	**↓**	**↓**	**↓**	*↓
Independent mother's income	**↑**	**↓**	**↑**	**↑**	**↑**
Mother herself breastfed	**↓**	**↓**	**↓**	**↓**	**↑**
Breast problems	**↑**	**↑**	**↑**	**↑**	**↓**
Maternal BMI <10th centile	**↑**	**↓**	**↓**	**↓**	**↓**
Maternal BMI >90th centile	**↓**	↑^*^	**↑**	**↑**	**↓**
Smoking during pregnancy	↓^*^	**↑**	↑^*^	**↑**	↑^*^
Pregestational dysthyroidism	**↓**	**↑**	**↑**	**↑**	**↑**
Severe pre-eclampsia	**↓**	**↑**	**↑**	↑^*^	**↓**
Maternal diabetes	**↑**	**↑**	**↑**	**↓**	**↓**
Maternal arterial hypertention	**↑**	**↑**	**↑**	**↑**	**↓**
Other maternal diseases	**↑**	↓^*^	**↓**	**↑**	**↑**
Assisted reproductive procedures	**↑**	**↑**	**↑**	**↑**	**↓**
Nulliparity	↓^*^	↑^*^	**↑**	**↑**	↑^*^
Gestational hypothyroidism	**↓**	**↑**	**↓**	**↓**	**↔**
**Peripartum confounders**					
Labor analgesia	**↓**	**↑**	**↑**	**↑**	**↑**
Cesarean delivery	**↓**	**↑**	**↑**	**↑**	**↓**
Weight gain <10th centile	**↓**	**↓**	**↓**	**↓**	**↓**
Weight gain >90th centile	**↓**	**↑**	**↓**	↑^*^	↑^*^
Male newborn sex	**↓**	**↑**	**↑**	**↑**	**↑**
Maternal fever (≥38°C)	**↓**	**↑**	**↓**	**↑**	**↑**
Post partum hemorrhage >1,000 mL	**↓**	**↑**	**↑**	**↑**	**↑**
Peripartum interventions	**↑**	↑^*^	**↓**	**↑**	**↓**
Labor induction	**↑**	**↑**	**↑**	**↑**	**↓**
Inhalation of meconium	**↑**	**↓**	**↓**	**↓**	**↑**
APGAR score at 5 min <7	**↑**	**↑**	**↑**	↑^*^	**↓**
Neonatal septic risk	**↓**	**↑**	**↓**	**↓**	**↑**
Neonatal hypoglycaemia	**↓**	↑^*^	**↑**	**↑**	**↓**
Neonatal phototherapy	**↓**	**↑**	**↑**	**↑**	**↑**
NICU admission	**↓**	↑^*^	**↓**	**↑**	**↑**
Skin-to-skin contact	**↓**	**↓**	**↓**	**↓**	**↑**
Rooming-in	**↑**	**↓**	**↓**	**↓**	**↑**

For each antelabor and peripartum confounder the effect on each endpoint (effect: **↑** favor or **↓** disadvantage) is indicated. Statistically significant effects are marked (^*^).

**Table 5:** Table of statistical significance.

	**Exclusive breastfeeding at 6 months**			**Integration**			**Complementary feeding**			**Exclusive formula feeding**			**Weaning**		
	**RR**	**95% CI**	***p*-value**	**RR**	**95% CI**	***p*-value**	**RR**	**95% CI**	***p*-value**	**RR**	**95% CI**	***p*-value**	**RR**	**95% CI**	***p*-value**
Weight Loss ≥7%	0.64	0.563–0.734	**0.000**	0.70	0.589–0.836	**0.000**	0.57	0.460–0.713	**0.000**	0.75	0.587–0.967	**0.026**	0.79	0.692–0.912	**0.001**
**Antelabor confounders**															
Maternal age <10th centile	0.89	0.676–1.168	0.398	1.38	0.971–1.961	0.073	1.30	0.796–2.109	0.298	0.87	0.539–1.418	0.586	0.78	0.577–1.042	0.092
Maternal age >90th centile	0.93	0.727–1.190	0.565	0.95	0.707–1.264	0.705	1.03	0.722–1.475	0.864	0.91	0.566–1.471	0.709	1.27	0.979–1.646	0.072
Paternal age <10th centile	0.98	0.780–1.234	0.870	1.23	0.918–1.643	0.166	1.09	0.700–1.709	0.694	1.02	0.671–1.541	0.938	0.85	0.668–1.085	0.194
Paternal age >90th centile	0.85	0.663–1.088	0.196	0.89	0.654–1.220	0.479	0.73	0.503–1.057	0.096	0.95	0.589–1.531	0.832	0.98	0.760–1.259	0.863
Mixed couple	0.91	0.738–1.123	0.381	0.79	0.590–1.046	0.099	0.77	0.539–1.103	0.155	0.88	0.523–1.490	0.641	1.00	0.801–1.245	0.992
Foreign couple	1.01	0.817–1.241	0.950	0.70	0.531–0.927	**0.013**	0.70	0.485–1.004	0.053	0.94	0.609–1.446	0.773	1.37	1.107–1.699	**0.004**
Lower maternal education	0.87	0.731–1.024	0.093	1.12	0.902–1.378	0.315	1.14	0.873–1.483	0.340	0.92	0.666–1.259	0.588	0.91	0.763–1.075	0.258
Lower paternal education	0.99	0.863–1.135	0.883	1.04	0.872–1.248	0.641	1.12	0.885–1.412	0.350	0.99	0.754–1.310	0.966	0.96	0.837–1.106	0.583
In-country couple residence	1.28	1.042–1.575	**0.019**	1.08	0.832–1.405	0.560	1.36	0.969–1.901	0.075	0.94	0.653–1.345	0.726	1.42	1.150–1.747	**0.001**
Independent mother's income	1.01	0.853–1.191	0.923	1.18	0.948–1.470	0.139	1.11	0.832–1.475	0.484	0.98	0.711–1.363	0.924	0.96	0.811–1.125	0.580
Mother herself breastfed	1.20	1.037–1.383	**0.014**	1.10	0.912–1.320	0.326	1.23	0.973–1.557	0.083	1.17	0.894–1.525	0.257	0.98	0.844–1.131	0.756
Breast problems	0.86	0.478–1.558	0.626	0.64	0.303–1.361	0.247	0.72	0.305–1.717	0.463	0.65	0.278–1.525	0.323	1.28	0.713–2.289	0.411
BMI <10th centile	0.94	0.766–1.164	0.589	1.00	0.744–1.342	0.997	0.98	0.675–1.422	0.914	0.96	0.616–1.500	0.861	0.99	0.799–1.224	0.919
BMI >90th centile	0.64	0.514–0.805	**0.000**	0.69	0.521–0.921	**0.012**	0.86	0.606–1.206	0.372	0.81	0.529–1.226	0.313	0.83	0.655–1.055	0.128
Smoking during pregnancy	0.80	0.641–0.991	**0.041**	0.80	0.603–1.063	0.125	0.67	0.460–0.964	**0.031**	0.76	0.528–1.101	0.148	0.77	0.620–0.961	**0.021**
Pregestational dysthyroidism	0.97	0.771–1.227	0.818	1.03	0.757–1.391	0.867	0.88	0.595–1.304	0.526	0.82	0.520–1.306	0.411	0.94	0.740–1.194	0.609
Severe pre-eclampsia	0.61	0.372–0.986	**0.044**	0.85	0.479–1.498	0.568	1.12	0.583–2.132	0.742	2.06	1.050–4.027	**0.036**	1.15	0.686–1.932	0.595
Maternal diabetes	0.96	0.759–1.217	0.739	1.00	0.729–1.381	0.984	0.91	0.619–1.340	0.634	0.91	0.545–1.520	0.720	0.99	0.787–1.255	0.957
Arterial hypertention	0.87	0.611–1.242	0.445	0.95	0.610–1.479	0.818	0.96	0..557–1 644	0 873	0 80	0 417–1 532	**0 500**	1 24	0.859–1 783	0 252
Other maternal diseases	0.82	0.618–1.083	0.160	0.62	0.426–0.910	**0.014**	0.67	0.412–1.078	0.098	0.73	0.423–1.265	0.263	0.97	0.734–1.294	0.858
Assisted reproductive procedures	0.60	0.381–0.937	**0.025**	1.18	0.678–2.067	0.554	0.56	0.289–1.069	0.079	1.20	0.485–2.970	0.693	0.98	0.614–1.566	0.936
Nulliparity	0.92	0.802–1.059	0.251	0.76	0.626–0.916	**0.004**	0.92	0.723–1.165	0.481	0.86	0.638–1.145	0.293	0.78	0.683–0.901	**0.001**
Gestational hypothyroidism	0.90	0.638–1.265	0.540	1.01	0.612–1.678	0..958	0.42	0.160–1.093	0.075	1.03	0.444–2.398	0.941	1.07	0.752–1.528	0.701

*For every antelabor confounder the effect on primary and secondary endpoints is indicated. P-value is in bold when the effect is statistically significant. RR, relative risk; CI, confidence interval*.

**Table 6:** Table of statistical significance.

	**Exclusive breastfeeding at 6 months**			**Integration**			**Complementary feeding**			**Exclusive formula feeding**			**Weaning**		
	**RR**	**95% CI**	***p*-value**	**RR**	**95% CI**	***p*-value**	**RR**	**95% CI**	***p-*value**	**RR**	**95% CI**	***p*-value**	**RR**	**95% CI**	***p*-value**
Weight Loss ≥7%	0.64	0.563–0.734	**0.000**	0.70	0.589–0.836	**0.000**	0.57	0.460–0.713	**0.000**	0.75	0.587–0.967	**0.026**	0.79	0.692–0.912	**0.001**
**Peripartum confounders**															
Analgesia in labor	0.92	0.531–1.575	0.748	0.90	0.716–1.119	0.331	1.04	0.776–1.390	0.798	0.83	0.600–1.146	0.257	0.95	0.800–1.135	0.589
Cesarean delivery	0.99	0.833–1.175	0.905	1.07	0.853–1.349	0.547	1.03	0.784–1.358	0.824	1.16	0.823–1.629	0.400	1.04	0.878–1.234	0.645
Weight gain <10th centile	1.11	0.840–1.456	0.474	1.13	0.747–1.715	0.558	0.94	0.527–1.660	0.819	1.14	0.591–2.213	0.691	1.01	0.756–1.346	0.953
Weight gain >90th centile	0.91	0.740–1.107	0.331	0.76	0.574–1.005	0.054	0.95	0.662–1.367	0.786	0.56	0.366–0.850	**0.007**	0.75	0.613–0.922	**0.006**
Male newborn sex	1.03	0.914–1.171	0.593	1.00	0.843–1.179	0.975	1.00	0.808–1.246	0.976	1.12	0.871–1.438	0.380	0.95	0.838–1.079	0.435
Maternal fever (≥38 °C)	1.83	0.968–3.474	0.063	1.65	0.777–3.521	0.191	1.16	0.317–4.266	0.820	0.59	0.171–2.053	0.408	0.60	0.314–1.133	0.114
Hemorrhage >1000 mL	1.03	0.755–1.406	0.849	1.11	0.739–1.666	0.615	0.64	0.393–1.025	0.063	1.11	0.620–1.986	0.726	1.04	0.765–1.419	0.794
Peripartum interventions	0.80	0.638–1.012	0.063	0.72	0.545–0.960	**0.025**	0.90	0.601–1.356	0.623	1.18	0.772–1.808	0.443	1.12	0.890–1.418	0.329
Labor induction	0.86	0.723–1.013	0.071	0.95	0.762–1.177	0.622	0.88	0.666–1.151	0.340	1.00	0.735–1.358	0.996	1.09	0.914–1.301	0.336
Meconium	1.02	0.882–1.171	0.826	1.13	0.934–1.361	0.213	1.05	0.829–1.341	0.667	1.26	0.955–1.671	0.102	0.93	0.812–1.072	0.327
APGAR score at 5 min <7	0.93	0.333–2.592	0.887	1.36	0.469–3.915	0.574	1.63	0.455–5.812	0.455	0.15	0.025–0.940	**0.043**	1.83	0.665–5.041	0.242
Neonatal septic risk	0.96	0.767–1.211	0.752	1.02	0.736–1.406	0.918	1.05	0.677–1.616	0.840	0.90	0.560–1.446	0.662	0.86	0.682–1.090	0.216
Neonatal hypoglycaemia	0.13	0.091–0.195	**0.000**	0.14	0.091–0.223	**0.000**	0.65	0.356–1.195	0.167	0.84	0.399–1.781	0.654	1.34	0.902–1.985	0.148
Phototherapy	0.67	0.431–1.043	0.076	0.63	0.381–1.049	0.076	0.57	0.290–1.109	0.097	0.97	0.403–2.334	0.945	1.08	0.682–1.696	0.756
NICU admission	0.40	0.256–0.622	**0.000**	0.33	0.199–0.548	**0.000**	2.69	0.934–7.731	0.067	0.93	0.339–2.568	0.894	0.89	0.569–1.400	0.621
Skin-to-skin contact	1.04	0.833–1.309	0.706	1.01	0.755–1.344	0.959	1.21	0.825–1.779	0.327	0.88	0.556–1.384	0.574	0.90	0.721–1.130	0.370
Rooming-in	1.28	1.009–1.631	**0.042**	1.21	0.897–1.623	0.214	0.95	0.637–1.429	0.820	1.19	0.742–1.904	0.472	1.14	0.889–1.470	0.296

*For every peripartum confounder the effect on primary and secondary endpoints is indicated. P-value is in bold when the effect is statistically significant. RR, relative risk; CI, confidence interval*.

**Figure 2:**
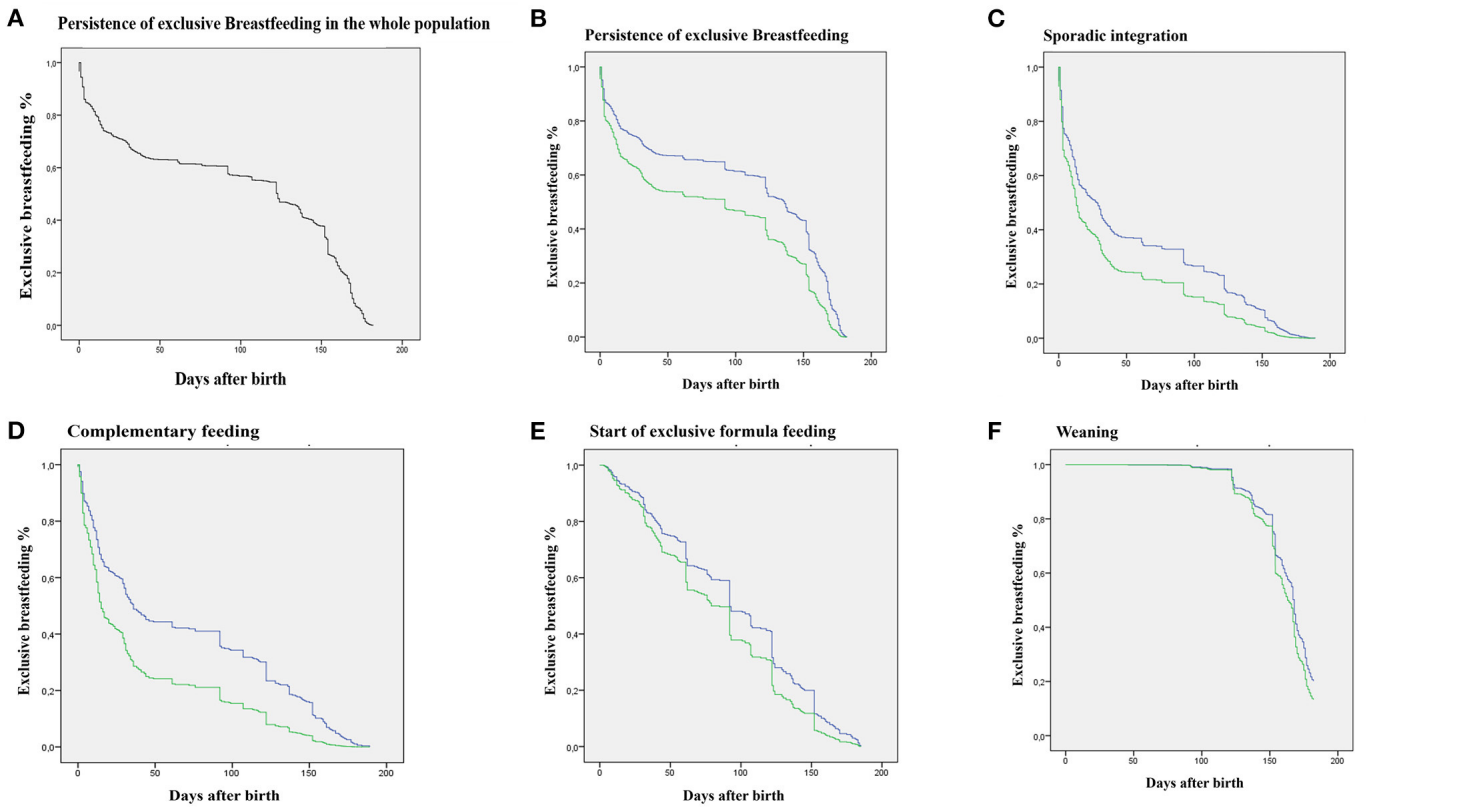
Survival curves obtained by Cox regression. Green: WL ≥7% group; blue: WL <7%. **(A)** Decay over time (days) of exclusive breastfeeding in the entire population. **(B)** Decay over time (days) of exclusive breastfeeding differentiated for the two groups. **(C)** Decay over time (days) of exclusive breastfeeding in relation to the occurrence of the event Sporadic Integration. **(D)** Decay over time (days) of exclusive breastfeeding in relation to the occurrence of the event Start of Complementary Feeding. **(E)** Decay over time (days) of exclusive breastfeeding in relation to the occurrence of the event Start of Exclusive Formula Feeding. **(F)** Decay over time (days) of exclusive breastfeeding in relation to the occurrence of the event Weaning. All curves, except the Weaning curve, significantly differ (*p* < 0.001).

By means of a Cox regression, a significantly different temporal course was observed between the two groups, for both time of interruption of exclusive breastfeeding and occurrence of sporadic integration, start of complementary feeding and start of exclusive formula feeding, but not for weaning (Figure 2).

## Discussion

We observed that WL ≥7% is significantly associated not only with a global reduction in the exclusive breastfeeding rate at 6 months, but also with all the four considered outcomes (i.e., increased frequency of sporadic integration, start of complementary feeding, shift to exclusive formula feeding, and weaning) up to 6 months. No one of the known described factors influencing exclusive breastfeeding at discharge has the same strong association at 6 months, significantly influencing only some of the four outcomes.

At our facility, certified as Baby Friendly Hospital (BFH) by UNICEF for 10 years in a row, we strictly follow the Ten Steps to Successful Breastfeeding, which are considered the optimal standard of in-hospital breastfeeding management. We observed that the average weight loss among healthy term exclusively breastfed newborns was 5.58%, inferior to that reported in other studies (20, 21), reflecting the importance of a breastfeeding supporting environment.

Additionally, only 31.3% of newborns in our study showed a WL ≥7%, similar to what has been reported by Bertini (22), whose hospital complied with the WHO guidance on infant feeding. Conversely, other researchers reported higher percentages, going as far as suggesting to raise the cut-off for physiological weight loss (21).

Breast problems only occurred in 15 of the 1,260 mothers enrolled, significantly contrasting with the incidence of mastitis (ranging from 2.5 to 20%) (23) and cracked nipples (24) published in previous studies. We can explain this interesting observation by specifying that in Aosta Valley women are followed extensively on the territory for a year after childbirth, by midwives trained on breastfeeding. Again, support seems to be crucial, even in preventing breast problems.

Interestingly, a WL ≥7% significantly impacts weaning when considering the absolute number of infants exclusively breastfed at 183 days from birth, yet survival curves describing the trend over time of the event “weaning” do not significantly differ. This discrepancy could be explained taking into account the incorrect but unfortunately widespread practice to start weaning in any case as early as 5 months. On the other hand, it seems to suggest an influence of weight loss on this same cultural factor: both the mother and health professionals may be less in a hurry to wean a baby with lower initial weight loss, thus reaching the goal of 183 days of exclusive breastfeeding.

Mode of delivery (cesarean delivery vs. vaginal delivery) does not influence persistence of exclusive breastfeeding at 6 months, as well as the other considered outcomes. We may infer that a strong breastfeeding support, offered by a multidisciplinary team (obstetricians, gynecologists, pediatricians, anesthetists, nurses) trained according to the UNICEF standards of the BFH project, can overcome the difficulties determined by the circumstances of birth (25).

In this regard, we observed that, consistently with current literature, in our cohort newborns from cesarean delivery lost significantly more weight than vaginally delivered newborns (6.18 vs. 5.39%), but average WL measured in our cohort was lower than that reported in the literature (26, 27).

It is interesting to note that authors describing major WL after cesarean delivery do not describe intraoperative fluid balance (26–28).

The observation that mode of delivery does not impact the persistence of exclusive breastfeeding at 6 months further reassures us on the validity of our intraoperative protocols, which consist in the administration of crystalloids (<100 ml per h of mother's preoperative fast before the umbilical cord clamping) and recommending mothers to drink clear fluids up to 4 h before surgery, thus avoiding excessive fluid administration during delivery. Limiting the maternal volume expansion may also protect from delayed lactogenesis II, which has been described after cesarean delivery (29).

We must also specify that at our hospital we weigh newborns for the first time from 2 to 6 hours after birth, that is after the initial period of diuresis, obtaining what Flaherman defines “the baseline dry weight” (30) and limiting bias related to intravenous fluid administration to the mother (31).

Among maternal illnesses, only severe preeclampsia seems to be related to the introduction of formula milk and start of exclusive formula feeding, perhaps because mothers in those conditions are often admitted to the intensive care unit and/or experience a delayed and less effective lactogenesis.

Being the only facility in the region is both a strength and a limit of our hospital: if on the one hand it allows us to study the entire population born in the region and ensure that every dyad of our cohort received the same standardized care, on the other hand it determines a population selection that could compromise the reproducibility of our results in other settings.

Additionally, the BFH designation of our hospital could have introduced a further selection, inducing foreign women to choose our facility to deliver. To control this selection bias, we included residence as a confounder in the multivariable analyses.

Another limit of our study is that the choice of considering, in the case of twins, only the first born, could introduce a bias, since a mother who has to care for two newborns cannot be as dedicated as one who only has one.

Finally, since the dates of occurrence of the events analyzed were provided by mothers in retrospect, they could be inaccurate, since a recall bias cannot be excluded.

However, the great sample size, the large number of confounders considered, and the long follow-up period may be considered the main strengths of our study.

In conclusion, a WL ≥7% in the first 72 h after birth is a simple and reproducible predictive factor for the early interruption of exclusive breastfeeding in healthy term exclusively breastfed newborns.

This parameter could aid the timely identification of dyads at risk of early interruption of exclusive breastfeeding, thus allowing health professionals to offer targeted care.

## Data Availability Statement

The raw data supporting the conclusions of this article will be made available by the authors, without undue reservation.

## Ethics Statement

The studies involving human participants were reviewed and approved by Ethics Committee from Azienda usl Valle d'Aosta. Written informed consent to participate in this study was provided by the participants' legal guardian/next of kin.

## Author Contributions

ED and RW contributed to conception and design of the study. RW and FV organized the database. LP performed the statistical analysis. ED, RN, and AC wrote the first draft of the manuscript. MG, MB, DM, FM, and LM wrote sections of the manuscript. All authors contributed to manuscript revision, read, and approved the submitted version.

## Funding

This research was received funding from University of Milan, Dipartimento Scienze Cliniche e di Comunità, for open access publication fees.

## Conflict of Interest

The authors declare that the research was conducted in the absence of any commercial or financial relationships that could be construed as a potential conflict of interest.

## Publisher's Note

All claims expressed in this article are solely those of the authors and do not necessarily represent those of their affiliated organizations, or those of the publisher, the editors and the reviewers. Any product that may be evaluated in this article, or claim that may be made by its manufacturer, is not guaranteed or endorsed by the publisher.

## Abbreviations

WL: Weight Loss
WHO: World Health Organization
UNICEF: United Nations Children's Fund
IBCLC: International Board Certified Lactation Consultant
BMI: Body Mass Index
BFH: Baby Friendly Hospital.

## References

[1] Theurich MA Davanzo R Busck-Rasmussen M Díaz-Gómez NM Brennan C Kylberg E. Breastfeeding rates and programs in Europe: a survey of 11 national breastfeeding committees and representatives. J Pediatr Gastroenterol Nutr. (2019) 68:400–7. 10.1097/MPG.0000000000002234 30562307

[2] Colombo L Crippa BL Consonni D Bettinelli ME Agosti V Mangino G. Breastfeeding determinants in healthy term newborns. Nutrients. (2018) 10:48. 10.3390/nu10010048 29304013 PMC5793276

[3] Odom EC Li R Scanlon KS Perrine CG Grummer-Strawn L. Reasons for earlier than desired cessation of breastfeeding. Pediatrics. (2013) 131:e726–32. 10.1542/peds.2012-1295 23420922 PMC4861949

[4] Dewey KG Nommsen-Rivers LA Heinig MJ Cohen RJ. Risk factors for suboptimal infant breastfeeding behavior, delayed onset of lactation, and excess neonatal weight loss. Pediatrics. (2003) 112:607–19. 10.1542/peds.112.3.607 12949292

[5] Noel-Weiss J Courant G Woodend AK. Physiological weight loss in the breastfed neonate: a systematic review. Open Med. (2008) 2:e99–e110. 21602959 PMC3091615

[6] American Academy of Pediatrics. Breastfeeding and the use of human milk policy statement. Pediatrics. (2005) 115:496–506. 10.1542/peds.2004-2491 15687461

[7] International Lactation Consultants Association. Clinical Guidelines for The Establishment of Exclusive Breastfeeding. Raleigh (NC): ILCA (2005). 10.1177/08903344241252646 38757900

[8] Goyal NK Attanasio LB Kozhimannil KB. Hospital care and early breastfeeding outcomes among late preterm, early-term, and term infants. Birth. (2014) 41:330–8. 10.1111/birt.12135 25294061 PMC12919936

[9] Campbell SH Lawers J Mannel R Spencer B. Core Curriculum for Interdisciplinary Lactation Care: Burlington, MA: Jones&Bartlett Learning (2019).

[10] De Rooy L Hawdon J. Nutritional factors that affect the postnatal metabolic adaptation of full-term small- and large-for-gestational-age infants. Pediatrics. (2002) 109:E42. 10.1542/peds.109.3.e42 11875170

[11] Rempel LA Rempel JK Moore KCJ. Relationships between types of father breastfeeding support and breastfeeding outcomes. Matern Child Nutr. (2017) 13:e12337. 10.1111/mcn.12337 27460557 PMC6865933

[12] Wallenborn JT Wheeler DC Lu J Perera RA Masho SW. Importance of familial opinions on breastfeeding practices: differences between father, mother, and mother-in-law. Breastfeed Med. (2019) 14:560–7. 10.1089/bfm.2019.0049 31298574

[13] Pettersson FD Hellgren C Nyberg F Åkerud H Sundström-Poromaa I. Depressed mood, anxiety, and the use of labor analgesia. Arch Womens Ment Health. (2016) 19:11–6. 10.1007/s00737-015-0572-6 26392364

[14] Almeida M Kosman KA Kendall MC De Oliveira GS. The association between labor epidural analgesia and postpartum depression: a systematic review and meta-analysis. BMC Womens Health. (2020) 20:99. 10.1186/s12905-020-00948-0 32393225 PMC7216422

[15] Heesen P Halpern SH Beilin Y Mauri PA Eidelman LA Heesen M. Labor neuraxial analgesia and breastfeeding: an updated systematic review. J Clin Anesth. (2021) 68:110105. 10.1016/j.jclinane.2020.110105 33069970

[16] French CA Cong X Chung KS. Labor epidural analgesia and breastfeeding: a systematic review. J Hum Lact. (2016) 32:507–20. 10.1177/0890334415623779 27121239

[17] World Health Organization, WHO Recommendations on Postnatal Care of the Mother And Newborn. (2013), ISBN: 978 92 4 150664 9 24624481

[18] Castillo H Santos IS Matijasevich A. Maternal pre-pregnancy BMI, gestational weight gain and breastfeeding. Eur J Clin Nutr. (2016) 70:431–6. 10.1038/ejcn.2015.232 26813940 PMC4827014

[19] Karall D Ndayisaba JP Heichlinger A Kiechl-Kohlendorfer U Stojakovic S Leitner H. Breast-feeding duration: early weaning-do we sufficiently consider the risk factors? J Pediatr Gastroenterol Nutr. (2015) 61:577–82. 10.1097/MPG.0000000000000873 26020371

[20] Davanzo R Cannioto Z Ronfani L Monasta L Demarini S. Breastfeeding and neonatal weight loss in healthy term infants. J Hum Lact. (2013) 29:45–53. 10.1177/0890334412444005 22554678

[21] DiTomasso D Paiva AL. Neonatal weight matters: an examination of weight changes in full-term breastfeeding newborns during the first 2 weeks of life. J Hum Lact. (2018) 34:86–92. 10.1177/0890334417722508 28800405

[22] Bertini G Breschi R Dani C. Physiological weight loss chart helps to identify high-risk infants who need breastfeeding support. Acta Paediatr. (2015) 104:1024–7. 10.1111/apa.12820 25283590

[23] Wilson E. Woodd Sl, Benova L. Incidence and risk factors for lctational mastitis: a systematic review. J Hum Lact. (2020) 36:673–86. 10.1177/0890334420907898 32286139 PMC7672676

[24] Milinco M Travan L Cattaneo A Knowles A Sola MV Causin E. Effectiveness of biological nurturing on early breastfeeding problems: a randomized controlled trial. Int Breastfeed J. (2020) 15:21. 10.1186/s13006-020-00261-4 32248838 PMC7132959

[25] Hutton EK Hannah ME Ross S Joseph KS Ohlsson A Asztalos EV. Maternal outcomes at 3 months after planned caesarean section vs. planned vaginal birth for twin pregnancies in the Twin Birth Study: a randomised controlled trial. BJOG. (2015) 122:1653–62. 10.1111/1471-0528.13597 26328526 PMC5014197

[26] Thulier D. Challenging expected patterns of weight loss in full-term breastfeeding neonates born by cesarean. J Obstet Gynecol Neonatal Nurs. (2017) 46:18–28. 10.1016/j.jogn.2016.11.006 27883879

[27] Preer GL Newby PK Philipp BL. Weight loss in exclusively breastfed infants delivered by cesarean birth. J Hum Lact. (2012) 28:153–8. 10.1177/0890334411434177 22526343

[28] Eltonsy S Blinn A Sonier B DeRoche S Mulaja A Hynes W. Intrapartum intravenous fluids for caesarean delivery and newborn weight loss: a retrospective cohort study. BMJ Paediatr Open. (2017) 1:e000070. 10.1136/bmjpo-2017-000070 29637114 PMC5862158

[29] Ilhan G Atmaca FV Çümen A Zebitay AG Güngör ES Karasu AFG. Effects of daytime vs. night-time cesarean deliveries on stage II lactogenesis. J Obstet Gynaecol Res. (2018) 44:717–22. 10.1111/jog.13562 29316014

[30] Flaherman VJ Schaefer EW Kuzniewicz MK Li S Walsh E Paul IM. Newborn weight loss during birth hospitalization and breastfeeding outcomes through age 1 month. J Hum Lact. (2017) 33:225–30. 10.1177/0890334416680181 28107100

[31] Chantry CJ Nommsen-Rivers LA Peerson JM Cohen RJ Dewey KG. Excess weight loss in first-born breastfed newborns relates to maternal intrapartum fluid balance. Pediatrics. (2011) 127:e171–9. 10.1542/peds.2009-2663 21173007

